# *De Novo* Design and Synthesis of Ultra-Short Peptidomimetic Antibiotics Having Dual Antimicrobial and Anti-Inflammatory Activities

**DOI:** 10.1371/journal.pone.0080025

**Published:** 2013-11-26

**Authors:** Ravichandran N. Murugan, Binu Jacob, Mija Ahn, Eunha Hwang, Hoik Sohn, Hyo-Nam Park, Eunjung Lee, Ji-Hyung Seo, Chaejoon Cheong, Ky-Youb Nam, Jae-Kyung Hyun, Ki-Woong Jeong, Yangmee Kim, Song Yub Shin, Jeong Kyu Bang

**Affiliations:** 1 Division of Magnetic Resonance, Korea Basic Science Institute, Ochang, Chung-Buk, Republic of Korea; 2 Department of Bio-Materials, Graduate School and Department of Cellular & Molecular Medicine, School of Medicine, Chosun University, Gwangju, Republic of Korea; 3 Department of Chemistry and Biochemistry, University of Texas at Austin, Austin, Texas, United States of America; 4 Division of Electron Microscopic Research, Korea Basic Science Institute, Daejeon, Republic of Korea; 5 Department of Bioscience and Biotechnology, Institute of SMART Biotechnology, Konkuk University, Seoul, Republic of Korea; 6 Bioinformatics and Molecular Design Research Center, Yonsei University Research Complex, Seoul, Republic of Korea; National Cancer Institute, NIH, United States of America

## Abstract

**Background:**

Much attention has been focused on the design and synthesis of potent, cationic antimicrobial peptides (AMPs) that possess both antimicrobial and anti-inflammatory activities. However, their development into therapeutic agents has been limited mainly due to their large size (12 to 50 residues in length) and poor protease stability.

**Methodology/Principal Findings:**

In an attempt to overcome the issues described above, a set of ultra-short, His-derived antimicrobial peptides (HDAMPs) has been developed for the first time. Through systematic tuning of pendant hydrophobic alkyl tails at the N(*π*)- and N(τ)-positions on His, and the positive charge of Arg, much higher prokaryotic selectivity was achieved, compared to human AMP LL-37. Additionally, the most potent HDAMPs showed promising dual antimicrobial and anti-inflammatory activities, as well as anti–methicillin-resistant *Staphylococcus aureus* (MRSA) activity and proteolytic resistance. Our results from transmission electron microscopy, membrane depolarization, confocal laser-scanning microscopy, and calcein-dye leakage experiments propose that HDAMP-1 kills microbial cells via dissipation of the membrane potential by forming pore/ion channels on bacterial cell membranes.

**Conclusion/Significance:**

The combination of the ultra-short size, high-prokaryotic selectivity, potent anti-MRSA activity, anti-inflammatory activity, and proteolytic resistance of the designed HDAMP-1, -3, -5, and -6 makes these molecules promising candidates for future antimicrobial therapeutics.

## Introduction

Antibiotics play an important role in preventing and treating diseases. However, excessive use of antibiotics has resulted in many emerging multidrug-resistant microorganisms; thus, antibiotic resistance has become a global public health problem. The emergence of multidrug-resistant bacteria and the low discovery rate of conventional antibiotics have resulted in an urgent need for developing new antimicrobials. Antimicrobial peptides (AMPs) are integral components of the innate host defense mechanism in many organisms, such as plants, insects, amphibians, and mammals. AMPs are generally 12 to 50 amino acids in length, contain excess positively charged amino acids (lysine and arginine residues) and around 50% hydrophobic amino acids, and fold into a diversity of amphipathic structures upon contact with microbial membranes [1]–[5]. They are emerging as a promising new generation of antibiotics because of their rapid and broad-spectrum antimicrobial properties, their ability to kill multidrug-resistant bacteria, and their low propensity for developing resistance [6], [7]. Although other mechanisms of action have been proposed, the bacterial killing effect of the majority of AMPs, such as melittin and LL-37, is considered to be because of their action on the lipid matrix of bacterial cell membranes, either by forming pores, thinning the membrane, or destabilizing the bilayer (i.e. the membrane-targeting AMPs) [8]–[10]. These mechanisms of action cause the lysis of bacterial cells as a result of increased permeability. In contrast, a few peptides, such as buforin-2 and PR-39, were known to penetrate microbial cell membranes without inducing membrane permeabilization, and cause bacterial cell death by inhibiting protein, DNA, or RNA synthesis (i.e. the intracellular-targeting AMPs) [11]–[13]. Moreover, AMPs also regulate the pro- and anti-inflammatory cytokine expression to modulate immune response by controlling the balance between inducing inflammation, and at the same time protecting the organism from the detrimental effects of excessive inflammatory response. For instance, apart from their direct antimicrobial activity, some cathelicidin-derived AMPs, including LL-37, CAP-18, SMAP-29, indolicidin, bactenecin, and β-defensin, are reported to have the ability to sequester lipopolysaccharide (LPS) and effectively neutralize its toxicity. This endotoxin is released from the bacteria during cell division or cell death, or, in particular, because of antibiotic therapy against bacterial infection [14]. Despite the many attractive properties of AMPs, their pharmaceutical development into therapeutic agents has been limited because of their large size, which poses several issues, including high production cost, poor protease stability, potential immunogenicity, and toxicity. A key strategy for resolving these problems is to design and synthesize shorter peptides with unusual amino acids, which capture the essential biological properties of AMPs. Several approaches are also being pursued to make shorter peptides [15]–[18], including cyclic peptides [19]–[21], non-natural peptidomimetics such as peptoids [4], [22]–[25], β-peptides [26]–[29], arylamide oligomers [30]–[31], γ-peptides [32], oligo-acyl lysines [33], [34], oligourea [35], and lipopeptides [36]–[38]. However, despite significant enthusiasm, there are intrinsic drawbacks associated with water solubility, complicated synthetic route, and difficulty of introducing a variety of functional groups to fine tune their activity and selectivity.

Therefore, in the present work, we focused on developing ultra-short AMPs (two or three residues in length) having dual antimicrobial and anti-inflammatory activities. A series of His-derived AMPs (HDAMPs) composed of one histidine (His) derivative with hydrophobic alkyl tails and one or two Arg residues were designed and synthesized. The solid-phase synthesis of HDAMPs is straightforward, using our His-derived amino acids, and they have a high potential for diversification at a relatively low cost. The prokaryotic selectivity of the designed HDAMPs was investigated by examining their antimicrobial activity against both Gram-positive and Gram-negative bacterial strains, and their hemolytic activity against human red blood cells (hRBCs). The anti-inflammatory activity of the designed HDAMPs was evaluated by examining inhibition of nitric oxide (NO) and tumor necrosis factor-α (TNF-α) production in LPS-stimulated mouse macrophage RAW 264.7 cells. The LPS-neutralizing activity of the designed HDAMPs was examined by the chromogenic *limulus* amebocyte lysate (LAL) assay. In order to identify the most promising, cost-effective ultra-short AMPs as a drug candidate, the designed HDAMPs were compared in terms of (i) broad-spectrum antimicrobial activity, (ii) prokaryotic selectivity, (iii) anti-inflammatory activity, (iv) anti-methicillin-resistant *Staphylococcus aureus* (MRSA) activity, and (v) protease stability. Finally, the molecular mechanism of antimicrobial activity was investigated by transmission electron microscopy (TEM), confocal laser-scanning microcopy, calcein-dye leakage, and membrane depolarization experiments. To the best of our knowledge, this is the first study that demonstrates effective and ultra-short HDAMPs (two or three residues in length) of antimicrobial agents having dual functions of antimicrobial and anti-inflammatory activities.

## Results

### Design and synthesis of Trp/Arg-rich and His-derived antimicrobial peptides (HDAMPs)

**Figure 1:**
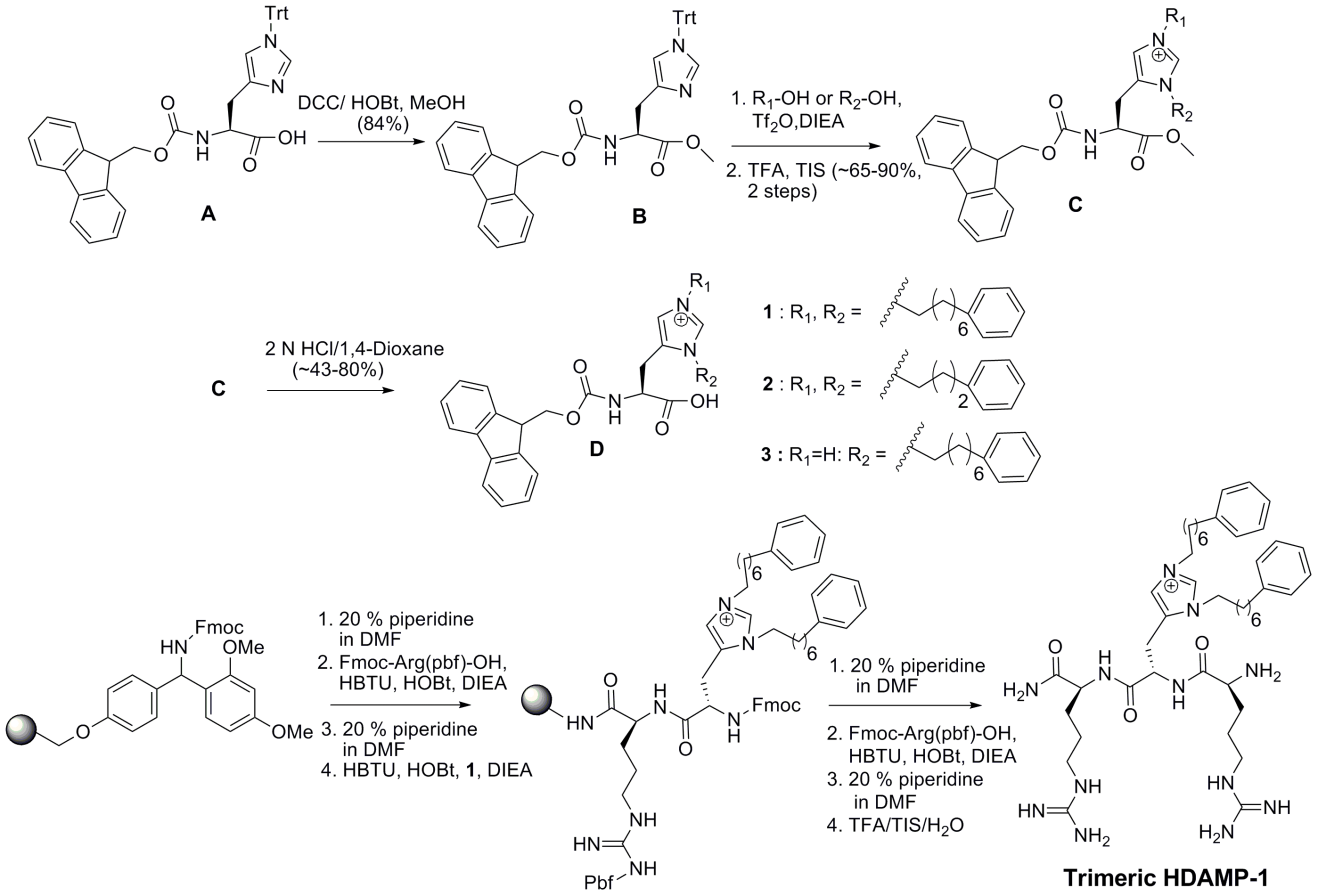
Synthetic protocol of Fmoc-His-OH derivatives and tripeptide HDAMP-1 using solid-phase peptide synthesis.

**Figure 2:**
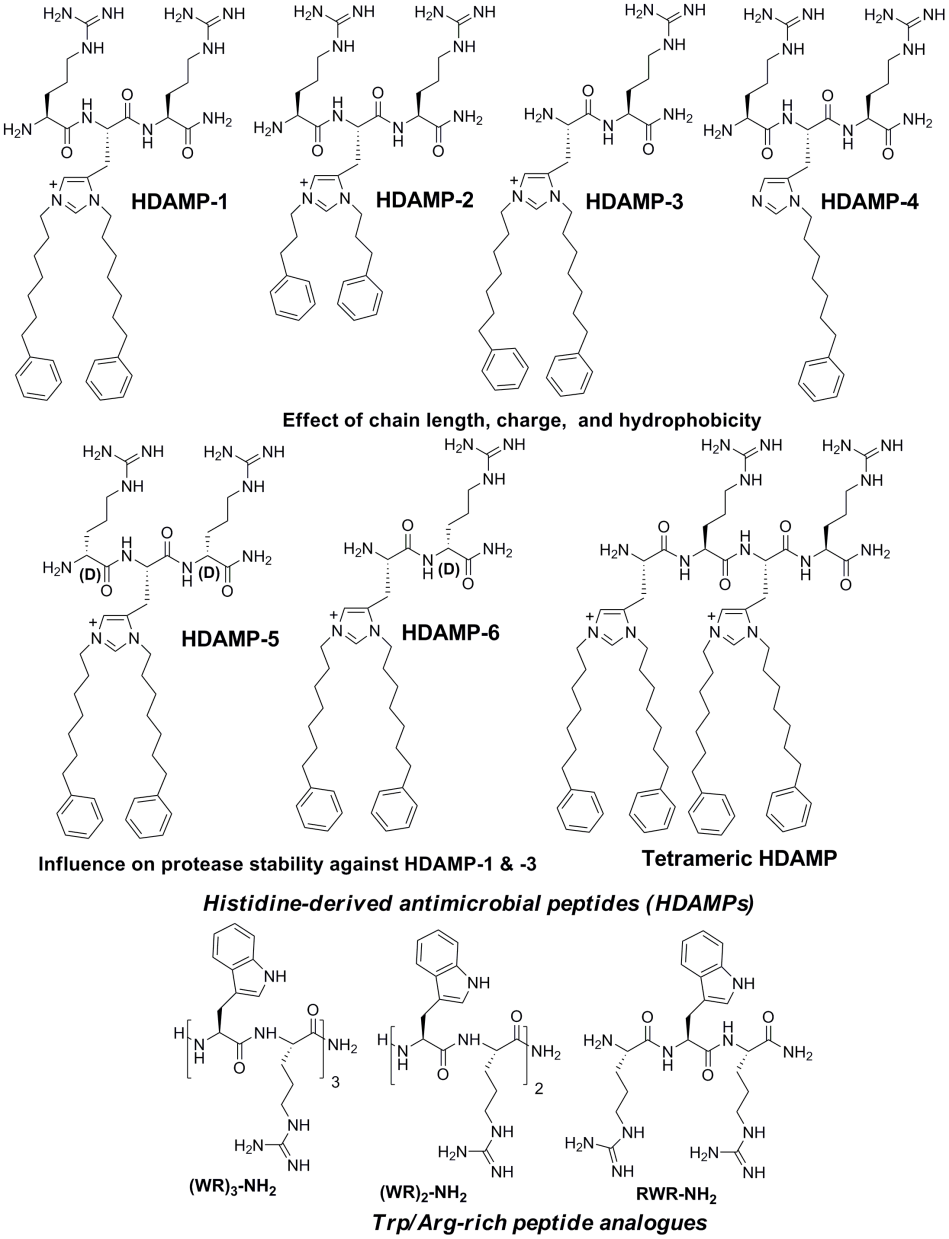
The structures of His-derived antimicrobial peptides (HDAMPs) and Trp/Arg-rich peptides were tested for their antimicrobial activity.

As a part of our continuing efforts to search for an inherently simple scaffold to pin down the importance of hydrophobicity and charge on antimicrobial activity, we have envisioned the use of His derivatives to capture the structural and biological properties of AMPs. A key feature in the design of these HDAMPs was the first-time isolation and purification of the Fmoc-protected His derivative, with pendant alkyl tails at the N(*π*)- and N(τ)- positions and bearing diverse structural variations, and the application of this derivative into the synthesis of antimicrobial peptides [39]. Briefly, the synthesis of Fmoc-protected His derivatives was preceded by a three-step synthesis method using commercially available Fmoc-His(Trt)-OH as a starting material. First, the esterification of Fmoc-His(Trt)-OH was efficiently carried out using dicyclohexylcarbodiimide (DCC) and 1-hydroxybenzotriazole (HOBt) as coupling agents in the presence of methanol. Second, the dialkylation at the N(*π*)- and N(τ)- positions on Fmoc-His(Trt)-OMe was proceeded using two equivalents of corresponding alcohol in the presence of triflic anhydride and DIEA. Finally, the acid hydrolysis of ester produced the expected Fmoc-protected, dialkylated His derivatives in good yield (Figure 1). Next, the above synthesized N(*π*)-alkyl and N(τ)-alkyl bis-adducted His monomers were successfully used to generate our focused peptide library, including seven HDAMPs using Rink amide resin by the conventional solid-phase peptide synthesis (Figure 1, Figure 2).

A series of Trp (W)/Arg (R)–rich peptides [(WR)_3_-NH_2_, (WR)_2_-NH_2_ and RWR-NH_2_], reported as being short and potent antimicrobial peptides [40], were used as sequence templates, and their antimicrobial and hemolytic activities were compared to seven HDAMPs (Figure 2). Matrix-assisted laser-desorption ionization time-of-flight mass spectrometry (MALDI-TOF MS) was used to verify the molecular weight of both Trp/Arg-rich AMPs and the seven HDAMPs. Human cathelicidin LL-37, a potent antimicrobial and anti-inflammatory peptide, served as a positive control for comparing the antimicrobial, hemolytic, and anti-inflammatory activities. The hydrophobicity of all of the peptides was measured with the use of reverse-phase high-performance liquid chromatography (RP-HPLC) retention time (R_t_).

### Antimicrobial and hemolytic activities and prokaryotic selectivity

All of the peptides were assayed for their antimicrobial activities towards two Gram-negative bacteria (*Escherichia coli and Pseudomonas aeruginosa*) and two Gram-positive bacteria (*Staphylococcus epidermidis and Staphylococcus aureus*). The prokaryotic selectivity of the peptides to mammalian cells was determined by measuring their hemolytic activity toward hRBCs, as described previously in our study (Table 1) [41].

**Table 1:** Antimicrobial and hemolytic activities and prokaryotic selectivity (therapeutic index) of designed HDAMPs.

Peptides	Minimal Inhibitory Concentration (MIC)^a^				GM^b^	% Hemolysis (HC_10_)^c^	TI^d^ (HC_10_/GM)
	*E. coli* [KCTC 1682]	*P. aeruginosa* [KCTC 1637]	*S. epidermidis* [KCTC 1917]	*S. aureus* [KCTC 1621]			
(WR)_3_-NH_2_	4 (3.8)	16 (15.3)	8 (7.7)	8 (7.7)	9 (8.6)	>256 (>245.1)	56.9
(WR)_2_-NH_2_	32 (45.6)	32 (45.6)	8 (11.4)	32 (45.6)	26 (37)	>256 (>364.5)	19.7
RWR- NH_2_	>64 (>124)	>64 (>124)	>64 (>124)	>64 (>124)	>64 (>124)	>256 (>495.8)	4.0
HDAMP-1	4 (4.9)	4 (4.9)	2 (2.5)	4 (4.9)	3.5 (4.3)	36 (44.1)	10.3
HDAMP-2	4 (5.7)	16 (22.7)	8 (11.4)	8 (11.4)	9 (12.8)	>256 (>363.9)	56.9
HDAMP-3	4 (6.1)	4 (6.1)	2 (3)	2 (3)	3 (4.6).	19 (28.8)	6.3
HDAMP-4	32 (49.9)	32 (49.9)	2 (3.1)	4 (6.2)	17.5 (27.3)	>256 (>399.1)	29.3
HDAMP-5	16 (19.6)	16 (19.6)	8 (9.8)	8 (9.8)	12.0 (14.7)	129 (158)	10.8
HDAMP-6	4 (6.1)	4 (6.1)	4 (6.1)	2 (3)	3.5 (5.3)	28 (42.4)	8
LL-37	32 (7.1)	32 (7.1)	64 (14.2)	16 (3.6)	36.0 (8)	167.5 (37.3)	4.7
Tetrameric HDAMP	200 (153.7)			200 (153.7)			

a Minimal inhibitory concentration of peptides in µg/mL; values in parentheses are in µM.

b The geometric mean (GM) of the MIC values against four bacterial strains in µg/mL; values in parentheses are in µM. When no detectable antimicrobial activity was observed at the maximum concentration (64 µg/mL), the value of twice the maximum concentration (128 µg/mL) was used to calculate the therapeutic index.

c HC_10_ is the peptide concentration that induces 10% hemolysis against human erythrocytes; in µg/mL; values in parentheses are in µM. When no detectable hemolytic activity was observed at the maximum concentration (256 µg/mL), the value of twice the maximum concentration (512 µg/mL) was used to calculate the therapeutic index.

d The therapeutic index was calculated as the ratio of the HC_10_ to the GM. Larger values of TI correspond to greater prokaryotic selectivity.

The therapeutic potential of antimicrobial agents lies in their prokaryotic selectivity to effectively kill bacterial cells without exhibiting significant cytotoxicity toward mammalian cells. The prokaryotic selectivity of our designed HDAMPs is defined by the therapeutic index (TI) as a measure of the relative safety of the drug. It is a measure of a peptide's capability to differentiate any pathogen from a host cell. The TI value of each peptide was calculated as the ratio of the HC_10_ (the peptide concentration that produces 10% hemolysis toward hRBCs) to the geometric mean [GM; the geometric mean of the minimal inhibitory concentration (MIC) against four selected bacterial strains]. Larger values of TI correspond to greater prokaryotic selectivity.

### Structure-antimicrobial and hemolytic activities studies

#### Correlation between Trp/Arg-rich AMPs and HDAMPs

The MICs obtained for the (WR)_3_-NH_2_ (4–16 µg/mL) was lower than that of LL-37, whereas the (WR)_2_-NH_2_, having two repeating WR units, showed an eight times decrease in antimicrobial activity; finally, the removal of one Trp unit from the (WR)_2_-NH_2_ (MIC:>64 µg/mL) at the N-terminal gave rise to a complete loss of antimicrobial activity. In contrast, all of the Trp/Arg-rich AMPs showed no hemolytic activity even at the highest concentration (256 µg/mL) tested. (Table 1, Figure 2).

Based on the results described above, we designed and synthesized both tripeptide and tetrapeptide HDAMPs (Arg-His((CH_2_)_7_C_6_H_5_)_2_-Arg-NH_2_) and His((CH_2_)_7_C_6_H_5_)_2_-Arg-His((CH_2_)_7_C_6_H_5_)_2_-Arg-NH_2_) (Figure 2), and tested the antimicrobial activity. Our assay results showed that the tetrameric HDAMP did not show any measurable antimicrobial activity at a concentration as high as 200 µg/mL (Table 1), whereas the shorter tripeptide, HDAMP-1, displayed one of the most potent short HDAMPs reported to date, with the MIC value of around 2–4 µg/mL against the bacterial strains (Table 1). Surprisingly, the activity of HDAMP-1 against *P. aeruginosa* and *S. epidermidis* was four times higher than the highly potent Trp/Arg-rich (WR)_3_-NH_2_ peptide. Although HDAMP-1 showed hemolytic activity, the TI (10.3) was two times higher than the TI of LL-37 (4.7; Table 1).

### Length of hydrocarbon tail

Next, to investigate the effect of the hydrocarbon tail length on antimicrobial activity, we decreased the length of the hydrocarbon tail from C-7 to C-3 (HDAMP-2). A small decrease (two- or four-fold) in the antimicrobial activity of HDAMP-2 was observed. However, HDAMP-2 displayed a higher TI value than HDAMP-1 because HDAMP-2 was non-hemolytic even at the high concentration of 256 µg/mL. This result suggests that the hemolytic activity (HC_10_ values) increased with an increase in the pendant alkyl tail length from C_3_ to C_7_ (Table 1, Figure S1).

### Charge

To understand the role of charge on HDAMPs in antimicrobial activity, we derived dipeptide HDAMP-3 by the N-terminal deletion of Arg and analyzed the antimicrobial activity. Encouragingly, dipeptide HDAMP-3 also displayed similar activity against Gram-negative bacteria, and it was even more active against Gram-positive, *S. aureus* bacteria in comparison to tripeptide peptide, HDAMP-1 (Table 1). This indicates that the lipophilicity of the pendant alkyl tails on HDAMPs was critical, and that the decreased cationic property resulting from the deletion of Arg at the N-terminus did not affect antimicrobial activity, but gave a two-fold decrease in the TI value due to the increased hemolytic activity (Table 1, Figure S1).

### Hydrophobicity

The higher activity and selectivity of HDAMP-1 and -3 could be because of the existence of a delicate balance between the charge density and hydrophobicity. This prompted us to further evaluate the effect of hydrophobicity independently of length by comparing dialkylated HDAMP-1 to N(*π*)-monoalkylated peptide, HDAMP-4 (Figure 2). The assay results showed that the hydrophobicity played an important role in antimicrobial activity against Gram-negative bacteria (*E. coli* and *P. aeruginosa)* because it showed an eight-fold decrease in activity when compared to HDMP-1. On the other hand, the activity towards the Gram-positive bacteria was not significantly affected (Table 1).

### Potency of D-Arg-substituted HDAMPs

D-amino acid forms of the peptides are well known for enhancing the stability and exploiting the specificity of proteases for L-amino acid forms. But the incorporation of D-amino acids in certain AMPs has caused considerable reduction in the activity when compared to the original L-amino acid peptide [42]. Considering the importance of our His-derived residues in providing potent antimicrobial activity, we synthesized the tripeptide HDAMP-5 and dipeptide HDAMP-6, in which the L-Arg of HDAMP-1 and HDAMP-3 was substituted by the corresponding D-form of Arg, respectively (Figure 2). HDAMP-5 showed a four-fold decrease in antimicrobial activity, but no hemolytic activity (HD_10_:>256 µg/mL, Figure S1). On the other hand, HDAMP-6 retained the antimicrobial activity of the original HDAMP-3, which had the L-amino acid, along with a slight increase in the prokaryotic selectivity (Table 1).

Overall, the results described above confirmed that the HDAMPs covered the broad spectrum of antimicrobial activity in comparison to at least Trp/Arg-rich AMPs (Table 1, Figure 2).

### Studies on anti-inflammatory activity

After establishing the potent antimicrobial activity using our HDAMPs, next, we investigated the anti-inflammatory activity. Here, we evaluated the anti-inflammatory activity of our designed ultra-short HDAMPs by examining the inhibition of NO and TNF-α production in LPS-stimulated RAW264.7 cells, as well as from the direct inhibition of LPS.

**Figure 3:**
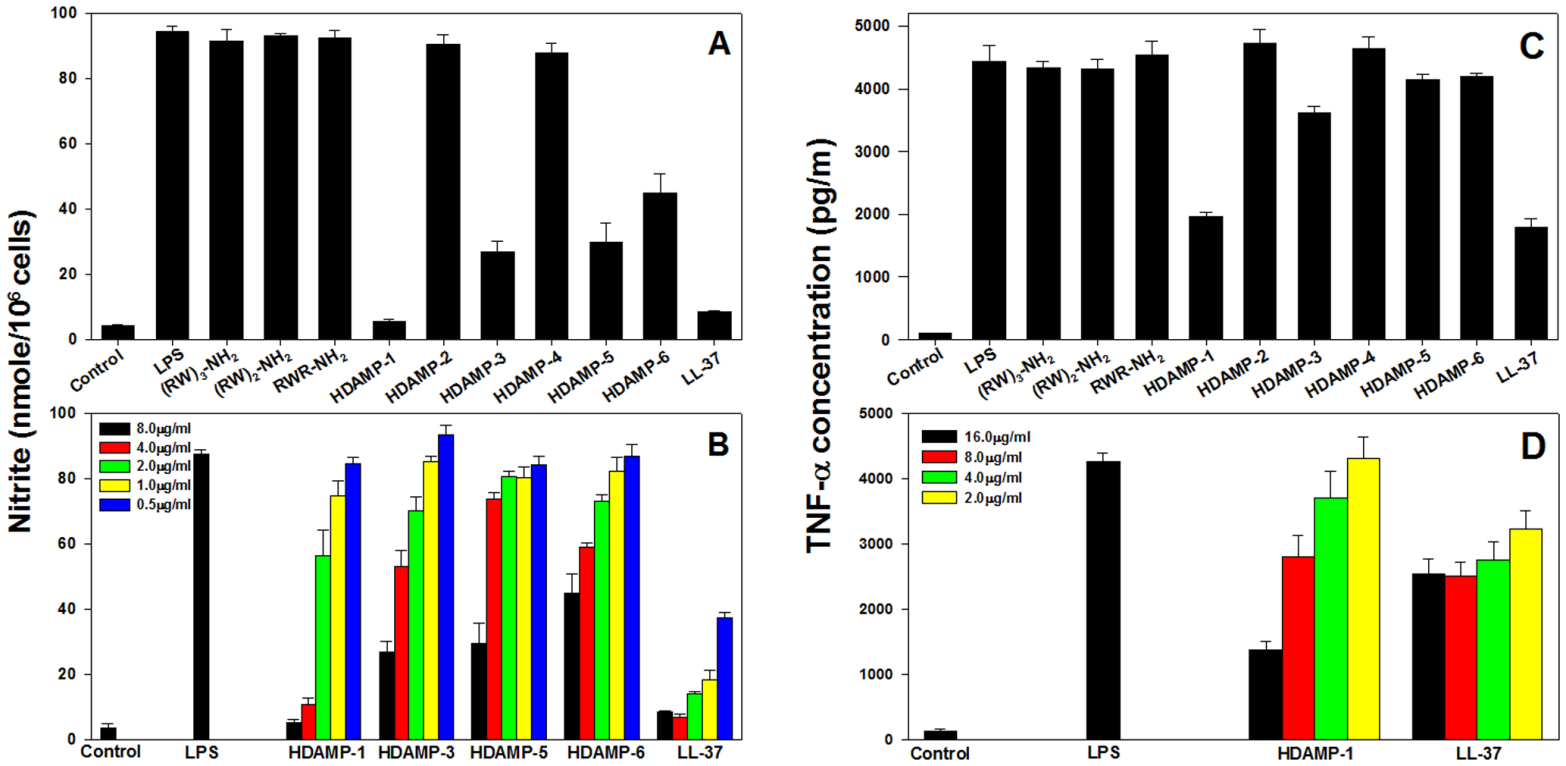
The inhibitory effect of the indicated peptides in RAW264.7 cells. Cells were stimulated with LPS (20 ng/mL). (*A–B*) Inhibitory activity against NO production in LPS-stimulated RAW264.7 cells. (*A*) Cells were treated with 8 µg/mL of each peptide. (*B*) Cells were treated with 0.5, 1, 2, 4, and 8 µg/mL of HDAMP-1, HDAMP-3, HDAMP-5, HDAMP-6, and LL-37. The cell-culture medium was collected, and the amount of nitrite released was measured. (*C–D*) The effects of the peptides on TNF-α release in LPS-stimulated RAW264.7 cells. (*C*) Cells were treated with 16 µg/mL of each peptide. (*D*) Cells were treated with 2, 4, 8, and 16 µg/mL of HDAMP-1 and LL-37. After incubation, the TNF-α concentration in the cell medium was evaluated using a mouse TNF-α ELISA kit. Data shown are representative of three independent experiments with similar results.

First, to compare the relative anti-inflammatory activity of the designed peptides, the inhibition assay for NO production in LPS-stimulated RAW264.7 cells was performed at the concentration of 8 µg/mL (Figure 3A). Among the designed ultra-short HDAMPs, HDAMP-1, -3, -5, and -6 significantly inhibited LPS-induced NO production. Encouragingly, HDAMP-1 showed similar inhibition of NO production compared to LL-37, a known naturally occurring AMP with potent anti-inflammatory activity. Moreover, as evidenced by the dose-dependent, LPS-induced NO production inhibition (Figure 3B), HDAMP-1 showed significant inhibitory activity at the concentration of 4 µg/mL, corresponding to its MIC value. In contrast, HDAMP-2 and -4 displayed no NO production inhibition at 8 µg/mL, when compared to HDAMP-1. Interestingly, one of the tripeptide HDAMP-5, differing in the isomeric variation of Arg as compared to HDAMP-1, showed 60% NO production inhibition in LPS-stimulated RAW264.7 cells (Figure 3B).

**Figure 4:**
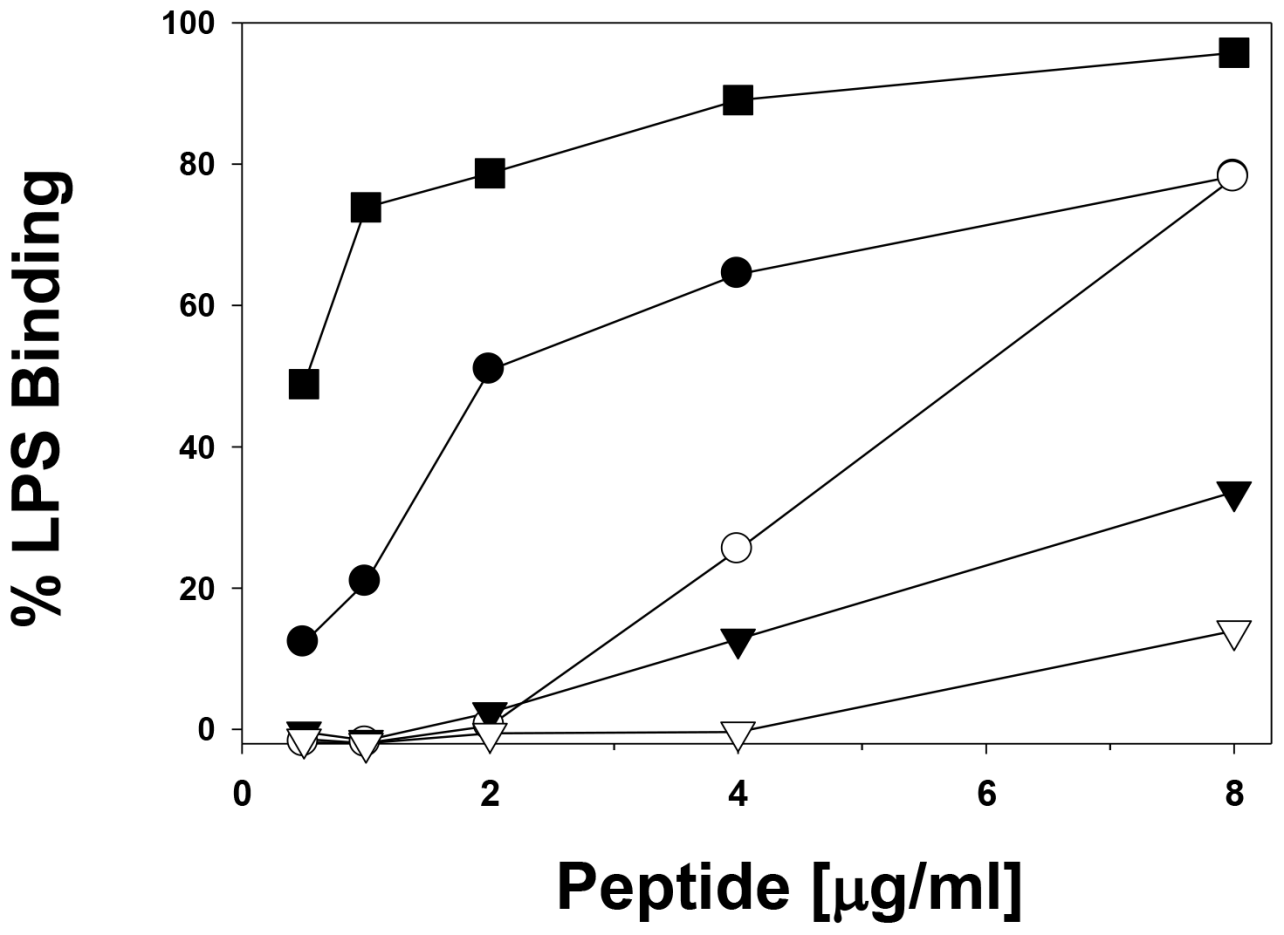
Concentration-response curves of LPS neutralization by the peptides, as determined by LAL assay. Peptides are indicated as follows: HDAMP-1 (•), HDAMP-3 (○), HDAMP-5 (▾), HDAMP-6 (▽), and LL-37 (▪).

A large body of evidence showed that TNF-α was the major mediator in endotoxic shock. We studied the inhibition of TNF-α released by LPS-stimulated macrophages in a dose-dependent manner using our HDAMPs and evaluated their endotoxin-neutralizing activity [43]. In contrast to NO production inhibition, only HDAMP-1 showed significant reduction of TNF-α release, which was similar to LL-37, whereas the other HDAMPs, such as 3, 5, and 6, were less active (Figure 3C). At the concentration of 16 µg/mL, HDAMP-1 increased the reduction of TNF-α release two-fold when compared to LL-37, whereas the 8 µg/mL of the peptide concentration was sufficient to exert a similar blockage of TNF-α by LL-37 (Figure 3D). Since the studies described above showed that HDAMP-1, -3, -5, and -6 effectively inhibited LPS-induced NO or TNF-α production in RAW264.7 cells, we asked whether these HDAMPs could directly block the binding of LPS. The concentration-response curve for LPS binding was evaluated in the presence of these HDAMPs and LL-37 by the LAL assay (Figure 4). The assay result showed that HDAMP-1 (•) and HDAMP-3 (○) had similar binding affinities when compared to LL-37 (▪), whereas HDAMP-5(▴) and -6 (Δ), having D-isoform of Arg, had little effect on LPS interactions (Figure 4). Consistent with this observation, these peptides did not significantly inhibit LPS-induced TNF-α production. Thus, the ability of the HDAMPs to inhibit LPS interactions depends on structural features in addition to their positive charges. Overall, the ability of different HDAMPs, such as 1, 3, 5, and 6, to inhibit LPS-stimulated NO or TNF-α production correlated well with their ability to block LPS binding.

### Antimicrobial activity against MRSA

The annual frequency of deaths from MRSA, a major cause of skin and soft-tissue infections in hospitals, is rapidly increasing and is greater than those caused by human immunodeficiency virus/acquired immune deficiency syndrome (HIV/AIDS) [44].

In addition to the broad-spectrum activity study described above, strains of MRSA (MRSA 1, 2, and 3) were employed as a multi-drug-resistant bacterium, for which susceptibility to a reference peptide, LL-37, and to the selected HDAMPs (1, 3, 5, and 6), having antimicrobial and anti-inflammatory activities, were tested. Surprisingly, our selected HDAMPs were found to cause a two-fold increase in activity against the methicillin-resistant strains than against the normal *S. aureus*, whereas LL-37 showed an increase in the level of MIC values (Table 2).

**Table 2:** Antimicrobial activity of designed HDAMPs against methicillin-resistant *Staphylococcus aureus* strains.

Peptides	Minimal Inhibitory Concentration (MIC)^a^		
	MRSA 1 [CCARM 3089]	MRSA 2 [CCARM 3090]	MRSA 3 [CCARM 3095]
HDAMP-1	2 (2.5)	2 (2.5)	2 (2.5)
HDAMP-2	16 (22.7)	32 (45.5)	16 (22.7)
HDAMP-3	2 (3)	2 (3)	2 (3)
HDAMP-4	16 (24.9)	16 (24.9)	16 (24.9)
HDAMP-5	8 (9.8)	8 (9.8)	8 (9.8)
HDAMP-6	2 (3)	2 (3)	2 (3)
LL-37	>32 (>7.1)	>32(>7.1)	>32(>7.1)

a Minimal inhibitory concentration of peptides in µg/mL; values in parentheses are in µM.

### Resistance to proteolytic degradation

**Figure 5:**
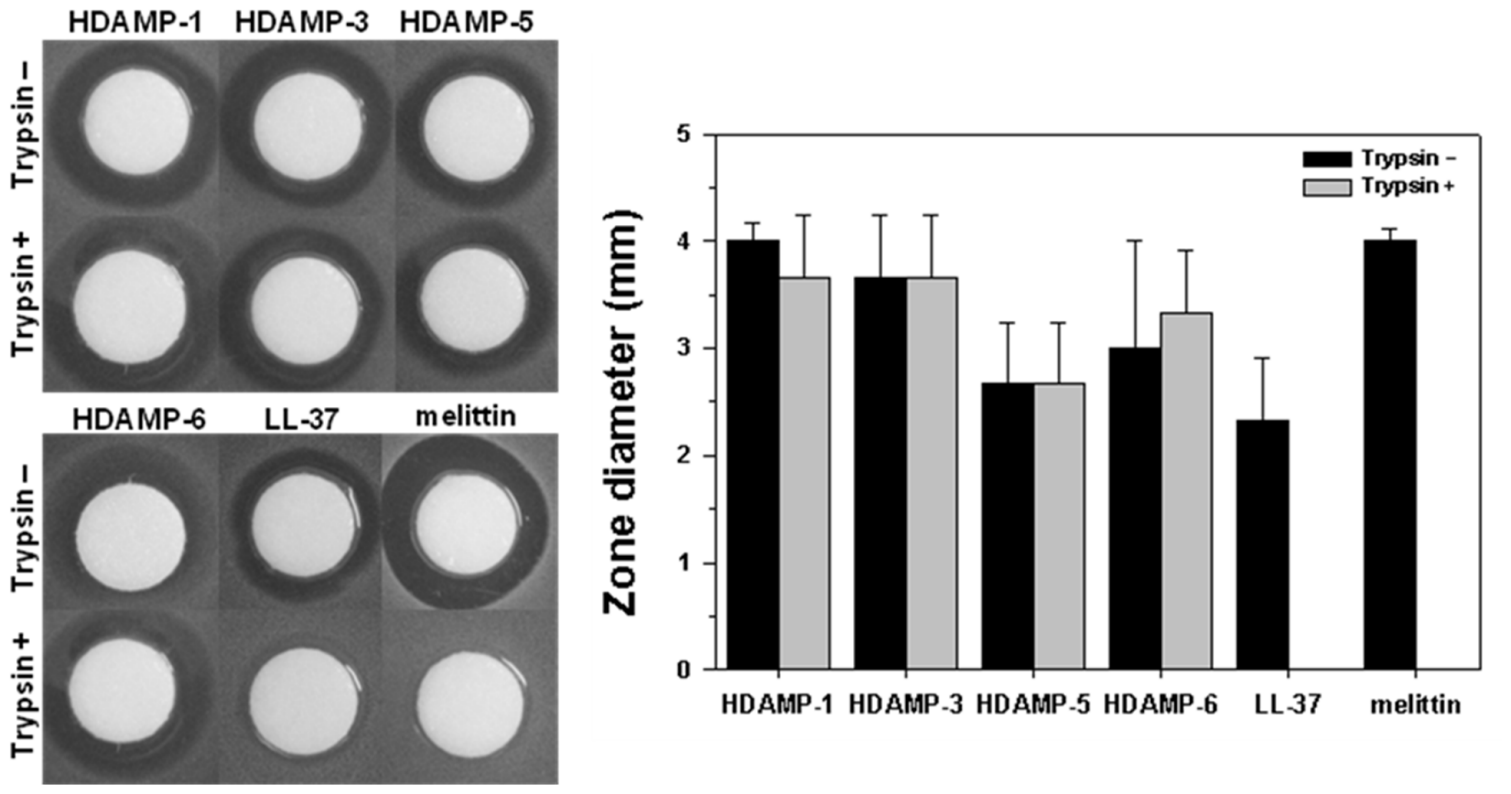
Inhibition of peptide antimicrobial activity against *E. coli* (KCTC 1682) by trypsin, assessed using the radial diffusion assay.

**Figure 6:**
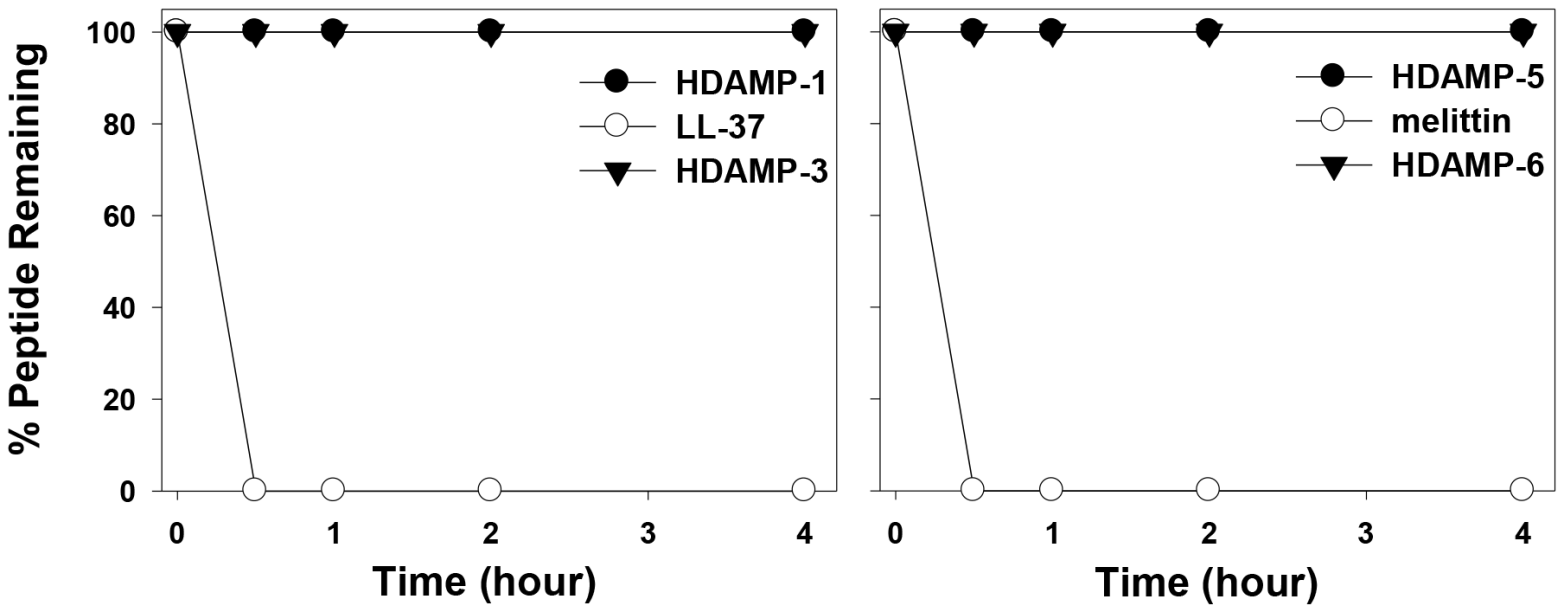
Proteolytic stability profiles of the designed HDAMPs, LL-37 and melittin, by trypsin. The relative concentrations of soluble peptides were analyzed by the integration of the absorbance at 224-HPLC chromatograms.

To further assess the potential of HDAMPs as drug candidates in peptide drug development, *in vitro* proteolytic stability has been investigated in human blood plasma using selected HDAMPs. Owing to the presence of several basic residues, AMPs are in fact highly susceptible to trypsin. As shown in Figure 5, HDAMPs 1, 3, 5, and 6 preserved their antimicrobial activity in the presence of trypsin after an incubation period of 24 h, while LL-37 and melittin (a cytotoxic AMP from bee venom) completely lost their antimicrobial activities. Additionally, the percentage of remaining peptides at each degradation time was examined using analytical RP-HPLC chromatograms after the trypsin treatment. Unlike melittin and LL-37, HDAMPs 1, 3, 5, and 6 were fully resistant to proteolysis by trypsin using RP-HPLC analysis (Figure 6).

### Antimicrobial mechanism study

Several AMPs have been extensively studied to elucidate their mode of antimicrobial action. To investigate the mechanism of action of the HDAMPs and the contribution of His derivatives with hydrophobic pendant alkyl tails on antibacterial activity, we performed the following detailed biochemical methods.

### Confocal laser-scanning microscopy

**Figure 7:**
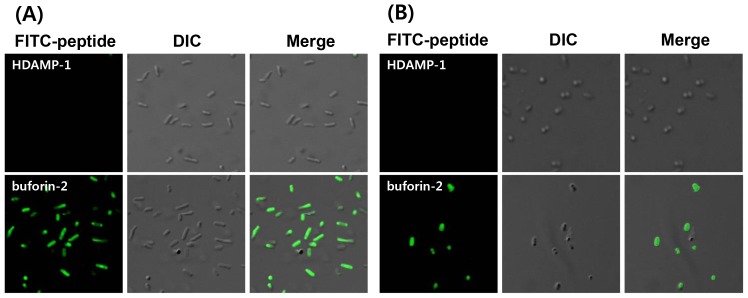
Confocal laser-scanning microscopic images of peptide-treated *E. coli* (KCTC 1682) or *S. aureus* (KCTC 1621) cells. (A) *E. coli* treated with FITC-labeled HDAMP-1 or buforin-2 at 5 µg/mL, and (B) *S. aureus* treated with FITC-labeled HDAMP-1 or buforin-2 at 5 µg/mL. For each fluorescence treatment, DIC and merge images have been presented.

To investigate the action site of HDAMP-1, fluorescein isothiocyanate (FITC)–labeled HDAMP-1 or buforin-2 was incubated with log-phase *E. coli* (KCTC 1682) or *S. aureus* (KCTC 1621), and their localization was visualized by confocal laser-scanning microscopy. As expected, buforin-2 penetrated the bacterial membrane without cell damage and accumulated in the cytoplasm of bacteria. In contrast, HDAMP-1 was unable to translocate the bacterial membrane. This finding indicates that the major site of action of HDAMP-1 is the bacterial membrane (Figure 7).

### Membrane depolarization

**Figure 8:**
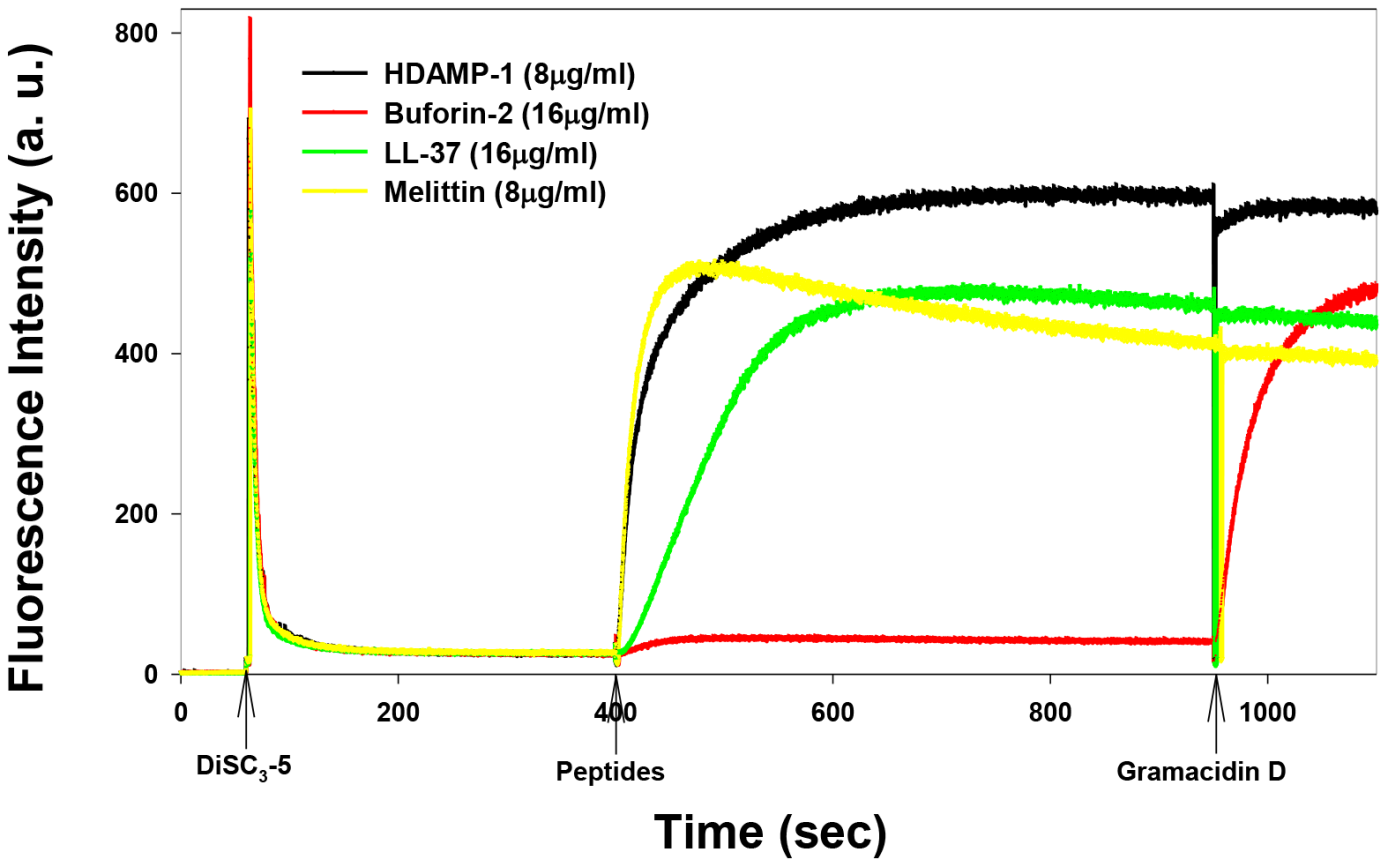
Time-dependent, peptide-induced cytoplasmic membrane depolarization against *S. aureus* (KCTC 1621, OD_600_ = 0.05). Membrane depolarization was measured by an increase in fluorescence of the membrane potential–sensitive dye, diSC_3_-5. Dye release was monitored at an excitation wavelength of 622 nm and an emission wavelength of 670 nm. In each run, the peptides were added near the 400-s mark.

To evaluate the effects of HDAMP-1 on *S. aureus* (KCTC 1621) cytoplasmic membranes, the membrane potential-sensitive fluorescent dye diSC_3_-5 was used. DiSC_3_-5 is distributed between the cells and medium, depending on the cytoplasmic membrane potential, and self-quenches when concentrated inside bacterial cells. If the membrane is depolarized, the probe will be released into the medium, causing a measurable increase in fluorescence. Similar to melittin and LL-37, HDAMP-1 induced a complete membrane depolarization under 2×MIC (8 µg/mL). In contrast, buforin-2 caused no or less membrane depolarization (Figure 8).

### Dye leakage

**Figure 9:**
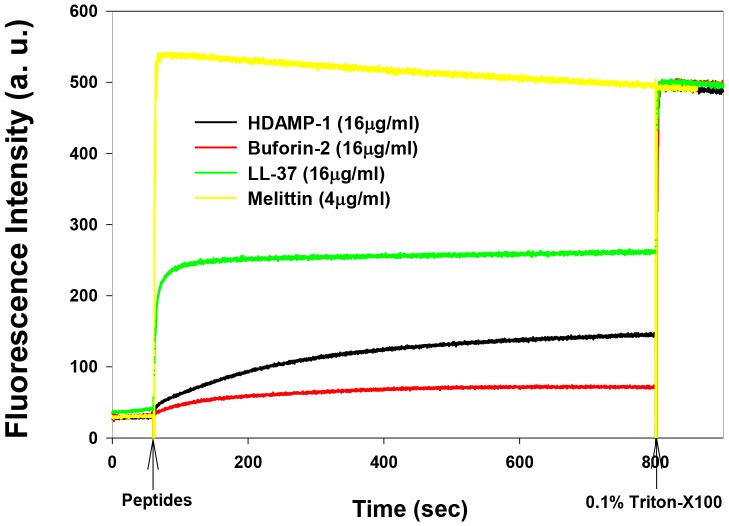
Time-dependent, peptide-induced dye leakage from calcein-entrapped, negatively charged PE/PG (7∶3, w/w) LUVs.

Next, the ability of HDAMP-1 to cause leakage of a fluorescent dye entrapped within the large unilamellar vesicles (LUVs) composed of negatively charged phosphatidylethanolamine (PE)/phosphatidylglycerol (PG) (7∶3, w/w) was tested. HDAMP-1 induced very low (less than 30%) calcein leakage at 16 µg/mL (Figure 9). This relatively lower calcein leakage from LUVs suggests that HDAMP-1 may act by forming pores, instead of rupturing the bacterial membrane.

### Transmission electron microscopy

**Figure 10:**
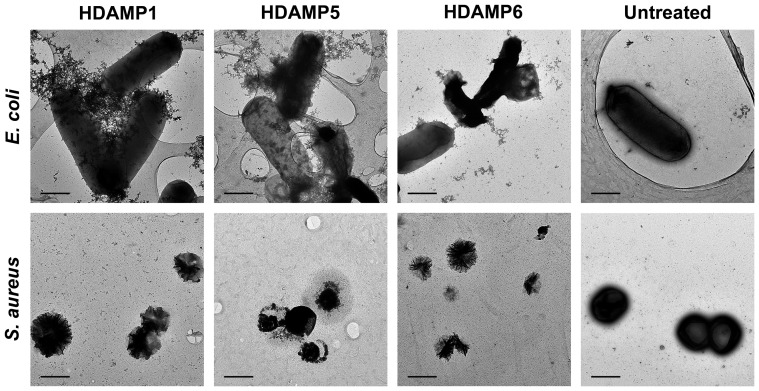
Electron micrographs of negatively stained *E. coli* and *S. aureus*, untreated or treated with HDAMPs. All images are scaled, and the scale bar represents 1 µm.

Finally, to study the morphological changes caused by the action of HDAMPs, *E. coli* and *S. aureus* were incubated with HDAMP-1, -5, and -6, and investigated by TEM. In comparison to the untreated control, an obvious change of the cellular structure was observed when HDAMPs were added (Figure 10). The release of internal materials was clearly visible by the treatment of HDAMPs, whereas the structures of untreated cells were preserved without any indication of cell damage.

## Discussion

Increased microbial drug resistance and the detrimental effects of an excessive inflammatory response have generated a global requirement for antimicrobial agents with dual functions.

In the present study, we focused on two areas. In the first part, we investigated how the shortest designed HDAMPs affected antimicrobial activity via systematic tuning of hydrocarbon tail length, charge, hydrophobicity, and stereo specificity. While this part was mostly a phenomenological description of the trends observed in the hemolytic and antimicrobial activities of these short, highly potent HDAMPs, the second section discussed the anti-inflammatory activity evaluated by examining HDAMP inhibition of NO, TNF-α production in LPS-stimulated RAW264.7 cells, and direct LPS neutralization. The second section also summarized the dual functionality of HDAMPs. Here, we utilized the two non-equivalent, but similarly reactive, nitrogen atoms on His, designed N(*π*) and N(τ), to generate the focused derivatives with pendant dialkylated tails, which was an effective method for obtaining both potent antimicrobial and anti-inflammatory activities. Notably, we have obtained the His-derived amino acids in high yield via a simple, three-step synthetic procedure (Figure 1).

Some of our designed HDAMPs exhibited potent broad-spectrum antimicrobial activity, with improved prokaryotic selectivity, and retained LPS neutralization activity in comparison to LL-37. In particular, HDAMP-1 showed promising antimicrobial activity (GM: 3.5 µg/mL), which was 10 times higher than LL-37 (GM: 36 µg/mL) and 10 times higher than the low HC_10_ (36 µg/mL) value against human erythrocytes. The results from the antimicrobial assay and hemolysis experiments indicate that by reducing the hydrophobicity of the peptide, the prokaryotic selectivity was enhanced as expected. Interestingly, HDAMPs (2, 4, and 5) were non-hemolytic and possessed higher therapeutic index values than the non-hemolytic LL-37 (Table 1). The structure–activity relationship (SAR) in this series of ultra-short peptides revealed that the maximum potencies and spectra of the antimicrobial activities correlated with the presence of one His, having a pendant alkyl tail (hydrophobicity), a lengthy carbon chain of pendant alkyl tails, an optimum number of Arg residues (cationicity), and stereospecificity (L-form preferred over D-Arg).

Strong evidence that HDAMP showed anti-inflammatory activity was obtained from the surprising inhibition of HDAMPs, such as 1, 3, 5, and 6, against NO, TNF-α production in the LPS-stimulated RAW 264.7 cells (Figure 3), and a mouse macrophage cell line. It is interesting to note the HDAMP-3, which has a higher hydrophobicity among HDAMPs, is less active than HDAMP-1. This difference in the anti-inflammatory activity led us to speculate that rather than global hydrophobicity, explicit involvement of the Arg stereochemical arrangement may be important for anti-inflammatory activity.

Finally, the HDAMPs, having the combination of antimicrobial and anti-inflammatory properties, were subjected to the anti-MRSA assay and trypsin-based proteolytic degradation to investigate the potential for their therapeutic application. Remarkably, the result of the anti-MRSA assay suggested that the selected shortest HDAMPs (1, 3, 5, and 6) overcame the development of MRSA resistance in comparison to LL-37 (Table 2). Moreover, the stability data clearly showed that our His derivatives, having hydrophobic pendant alkyl tails, gave the HDAMPs greater protease stability beyond the additive contribution of the individual D-Arg amino acids (Figures 5 and 6). In addition, based on the mechanistic studies (Figures 7–10), we propose here that HDAMP-1 kills microorganisms via dissipation of the membrane potential by forming pore/ion channels on bacterial cell membranes (barrel stave model or toroidal model), and not via the membrane-disruption/perturbation modes (carpet-like model). Although many problems still need to be solved, the undesirable properties of AMPs that stem from MRSA resistance, protease stability, and immunogenicity could be overcome with these ultra-short HDAMPs.

We demonstrated the first synthesis of the shortest HDAMPs, having diverse structural scaffolds obtained by facile chemical tailoring, which provides potent antimicrobial and anti-inflammatory activities, as well as enhanced bioavailability due to high water solubility and protease stability. Some of our designed ultra-short HDAMPs (1, 3, 5, and 6) exhibited not only high antimicrobial activity against Gram-positive and Gram-negative bacterial strains, including MRSA, with no or less hemolytic activity, but also significantly inhibited NO and/or TNF-α release in LPS-stimulated immune cells and neutralized LPS. Moreover, we showed the systematic comparison of common structural parameters of HDAMPs, such as charge, hydrophobicity, and length, which are important for the comprehensive understanding of how peptides kill pathogens. Considering all these things, our HDAMPs have given us a new strategy for overcoming the main hurdles of AMPs, including chain length, limited stability, solubility, MRSA resistance, and mass production, which have limited their development into therapeutic agents.

## Materials and Methods

Rink amide 4-methylbenzhydrylamine (MBHA) resin and 9-fluorenylmethoxycarbonyl (Fmoc) amino acids were obtained from Calbiochem-Novabiochem (La Jolla, CA). Other reagents used for peptide synthesis included trifluoroacetic acid (TFA; Sigma), piperidine (Merck), dicyclohexylcarbodiimide (DCC; Fluka), 1-*O*-Benzotriazole-*N*,*N*,*N*′,*N*′-tetramethyl-uronium-hexafluoro-phosphate (HBTU; Aldrich), 1-hydroxybenzotriazole (HOBT; Aldrich), *N*,*N*-diisopropylethylamine (DIEA; Aldrich) and dimethylformamide (DMF, peptide synthesis grade; Biolab). Lipopolysaccharide (LPS, from *E. coli* O111:B4) and 3-(4,5-dimethylthiazol-2-yl)-2,5-diphenyl-2*H*-tetrazolium bromide (MTT) were supplied from Sigma Chemical Co (St. Louis, MO). DMEM and fetal bovine serum (FBS) were obtained by HyClone (Seoulin, Bioscience, Korea). RAW264.7 cells were purchased from American Type Culture Collection (Bethesda, MD). Phosphatidylethanolamine (PE, from egg yolk), phosphatidylglycerol (PG, from egg yolk), and calcein were purchased from Sigma (St. Louis, MO). DiSC_3_-5 was obtained from Molecular Probes (Eugene, OR). All other reagents were of analytical grade. The buffers were prepared in double glass–distilled water.

### Synthesis and characterization of HDAMPs

All peptides were prepared by Fmoc SPPS methods using Rink amide with an initial loading of 0.61 mmol/g, unless otherwise noted. Fmoc-Arg(Pbf)-OH and other Fmoc-protected amino acids were purchased from Novabiochem. Resins were swollen in DMF for 45 min prior to synthesis. For sequence extension, the Fmoc-protected amino acid (5 eq.) was activated by treatment with HBTU (5.0 eq.), 1-hydroxybenzotriazole (HOBt; 5.0 eq.), and DIEA (10 eq.) in DMF (2 mL) for 2 min. This solution was added to the free amine on resin, and the coupling reaction was allowed to proceed for 1 h with vortex stirring. After washing with DMF, Fmoc de-protection was achieved with 20% piperidine in DMF (1×10 min, 2×3 min). The resin was washed once again, and the process was repeated for the next amino acid; finally, the resin was washed with DMF, methanol, dichloromethane, and ether, and then dried under a vacuum. In the case of FITC-labeled peptides, Fluorescein 5-isothiocyanate (2 eq.) was added in DMF, along with the DIEA (4 eq.), to the N-terminal free amine on resin, and the coupling was allowed to proceed overnight with vortex stirring. All the peptides were cleaved from the resin with 5% triisopropylsilane (TIS) and 5% H_2_O in trifluoroacetic acid (TFA, approximately 2 mL of TFA per 100 mg of resin) for 2 h. The cleavage mixture was mixed with cold ether to precipitate the peptide, and then filtered. RP-HPLC analysis was carried out on the prepared Vydac C_18_ column (15 µm, 20 mm ×250 mm), using an appropriate 0−90% water/acetonitrile gradient in the presence of 0.05% TFA. The final purity and hydrophobicity of the peptides (>98%) were assessed by RP-HPLC on an analytical Vydac C_18_ column (4.6 mm ×25 0 mm, 300 Å, 5 µm particle size, Table 3). The molecular masses of purified peptides were determined using MALDI-TOF MS (Shimadzu, Japan).

**Table 3:** RP-HPLC retention time of the indicated HDAMPs.

Peptides	RP-HPLC retention time (R_t_): min
HDAMP-1	29.21
HDAMP-2	21.32
HDAMP-3	31.05
HDAMP-4	21.77
HDAMP-5	28.91
HDAMP-6	30.78

RP-HPLC retention time (R_t_) was measured using a C_18_ reverse-phase analytical column (5 µm; 4.6 mm ×250 mm; Vydac). Peptides were eluted for 60 min using a linear gradient of 0–90% (v/v) acetonitrile in water containing 0.05% (v/v) trifluoroacetic acid.

### General procedure for the preparation of N (*α*)-[(9*H*-Fluoren-9-ylmethoxy)carbonyl]-N(*π*)-,N(τ)-bisalkyl-L-histidine. The synthesis was performed in accordance with Qian et al [39]

Compound B: (Figure 1)

A 100-mL Schlenk flask was charged with solid Fmoc-His(Trt)-OH (10 g, 16.14 mmol) and HOBt (3.27 g, 24.20 mmol), and was degassed by vacuum-Ar purge cycle. Dry THF (80 mL) was added to dissolve all solids, and the solution was cooled to -13°C with an ice-acetone bath. A dry THF solution (80 mL) of DCC (3.33 g, 16.14 mmol) was added to this mixture dropwise, along with MeOH, (12.40 mL, 513.0 mmol). The reaction was allowed to warm slowly to room temperature while stirring overnight, during which time solid dicyclohexylurea was observed to precipitate. This was removed by vacuum filtration through a medium-porosity frit, and the solvent was removed by rotary evaporation. The oily residue was re-dissolved in dichloromethane, washed three times with aqueous NaHCO_3_, and washed three times with water, and the organic layer was dried over MgSO_4_. The solvent was removed by rotary evaporation, and a crude yellow solid was isolated after drying. The compound was purified by column chromatography on silica gel and eluted with 1% MeOH-DCM (10.34 g, quantitative yield) R_f_ = 0.33

^1^H-NMR (300 MHz, CDCl_3_): δ 7.75 (d, 2H, *J* = 7.5 Hz), 7.62 (t, 2H, *J* = 7.5 Hz), 7.53 (s, 1H), 7.42–7.26 (m, 13H), 7.13–7.10 (m, 6H), 6.57 (s, 1H), 6.53 (d, 1H, *J* = 8.2 Hz), 4.63 (m, 1H), 4.38–4.22 (m, 3H), 3.63 (s, 3H), 3.13–3.03 (m, 2H). All the values are in agreement with the reported values [45].

### Synthesis of N (*α*)-[(9*H*-Fluoren-9-ylmethoxy)carbonyl]-N(*π*)-,N(τ)-bis(7-phenylheptyl)-L-histidine methyl ester (Compound C1)

A solution of 7-phenyl-1-heptanol (1.12 mL, 5.58 mmol) and diisopropylethylamine (DIEA) (973 µL, 5.58 mmol) in DCM (50 mL), added dropwise over 10 min, was added to a stirred solution of triflic anhydride (939 µL, 5.58 mmol) in DCM (35 mL) under Ar at −75°C. Stirring was continued at −75°C (20 min); then, a solution of FmocHis(Trt)-OMe (1.77 g, 2.79 mmol) in DCM (25 mL) was added dropwise, and the mixture was allowed to gradually warm to room temperature over a period of 18 h. The mixture was quenched using aqueous NaHCO_3_ and stirred vigorously (30 min). The organic layer was diluted with DCM and washed with aqueous NaHCO_3_ and brine, then dried (MgSO_4_), and concentrated to viscous oil. Trifluoroacetic acid (2 mL, 27.90 mmol) and TIS (629 µL, 3.07 mmol) were added to a solution of the resulting gum in DCM (25 mL), and the mixture was stirred at room temperature until the reaction was complete, as shown by TLC (2 h). The solvent was removed in vacuo, and the residue was purified by silica gel column chromatography using 1–5% MeOH in DCM to provide the product as a pale, yellow gum (1.73 g, 85% yield). Note that although the *in situ*–generated triflates will remove the trityl-protecting group from the histidine, we treated again with TFA in order to make sure the trityl group was completely removed from either the minor monoalkylated or unknown product, for a better purification process.

^l^H-NMR (300 MHz, CDCl_3_): δ 8.91 (s, 1H), 7.77 (d, *J* = 7.4 Hz, 2H), 7.57–7.68 (m, 2H), 7.23–7.08 (m, 14H), 6.23 (br. s, 1H), 4.60 (m, 1H), 4.37 (d, *J* = 6.0 Hz, 2H), 4.21 (t, *J* = 6.9 Hz, 1H), 3.99–4.15 (m, 4H), 3.78 (s, 3H), 3.17–3.30 (m, 3H), 2.53–2.67 (m, 4H), 1.69–1.78 (m, 3H), 1.47–1.66 (m, 5H), 1.38–1.47 (m, 3H), 1.23 (s, 9H).

### Synthesis of N (*α*)-[(9*H*-Fluoren-9-ylmethoxy)carbonyl]-N(*π*)-,N(τ)-bis(7-phenylheptyl)-L-histidine (Compound D1)

The 1∶1 mixture of 2 N HCl (50 mL) and 1,4-Dioxane (50 mL) solution was added to the C1 (1.6 g, 2.20 mmol), and reflux occurred at 100°C for 3 h. The mixture was brought to room temperature, and the solvent was removed by Rota vapor. The resulting aqueous mixture was extracted with DCM, washed with brine, dried (MgSO_4_), and concentrated to viscous oil. The organic extract was dried (MgSO_4_) and concentrated in vacuo, and the residue was purified by silica gel–flash chromatography from 5% to 20% MeOH in DCM to provide the acid as a light yellow gum (0.69 g, 43% yield).

^l^H-NMR (300 MHz, CDCl_3_): δ 8.47 (s, 1H), 7.72 (d, *J* = 7.4 Hz, 2H), 7.52–7.68 (m, 2H), 7.08–7.43 (m, 14H), 6.83 (m, 1H), 4.34 (m, 1H), 4.03–4.28 (m, 3H), 3.82–4.03 (m, 2H), 3.49–3.73 (m, 2H), 2.98–3.33 (m, 2H), 2.35–2.72 (m, 4H), 1.59–1.89 (m, 4H), 1.41–1.59 (m, 4H), 1.21 (s, 12H).

^13^C-NMR (75 MHz, CDCl_3_): δ 172.5, 156.2, 143.9, 143.3, 142.6, 142.5, 141.3, 141.2, 134.6, 131.2, 128.4, 128.3, 127.9, 127.2, 125.7, 125.3, 125.0, 121.3, 120.0, 67.0, 53.6, 49.9, 47.3, 46.9, 35.8, 31.2, 31.1, 29.9, 29.8, 28.9, 28.7, 28.6, 26.2, 26.0, 24.8.

MS (MALDI-TOF) m/z 726.43[M]^+^.

### Synthesis of N (*α*)-[(9*H*-Fluoren-9-ylmethoxy)carbonyl]-N(*π*)-,N(τ)-bis(3-phenylpropyl)-L-histidine methyl ester (Compound C2)

The title compound was synthesized in a manner identical to compound C1, using 3-phenylpropanol (709 µL, 5.21 mmol) instead of 7-phenyl-1-heptanol. Materials: Fmoc-His(Trt)-OMe (1.5 g, 2.37 mmol, 20 mL DCM), Tf_2_O (877 µL, 3.16 mmol, 35 mL DCM), DIEA (911 µL, 5.21 mmol, 45 mL DCM), TFA (1.8 mL, 23.70 mmol), and TIPS (534 µL, 2.61 mmol, 20 mL DCM). The compound is purified by column chromatography on silica gel, eluted with 1–5% MeOH-DCM. 1.33 g, 89% yield.

^l^H-NMR (300 MHz, CDCl_3_): δ 8.91 (s, 1H), 7.80 (d, *J* = 7.4 Hz, 2H), 7.56–7.70 (m, 2H), 7.04–7.49 (m, 14H), 6.63 (d, *J* = 7.6 Hz, 1H), 4.49 (m, 1H), 3.97–4.39 (m, 7H), 3.76 (s, 3H) 3.15 (s, 2H), 2.70 (t, *J* = 7.14 Hz, 2H), 2.59 (t, *J* = 7.1 Hz, 2H), 2.20–2.04 (m, 4H).

### Synthesis of N (*α*)-[(9*H*-Fluoren-9-ylmethoxy)carbonyl]-N(*π*)-,N(τ)-bis(3-phenylpropyl)-L-histidine (Compound D2)

The title compound was synthesized in a manner identical to compound D1, using C2 instead of C1 (1.33 g, 2.11 mmol). Materials: 2 N HCl: 1,4-Dioxane (45 mL∶ 45 mL). The compound was purified by column chromatography on silica gel, eluted with 5–20% MeOH-DCM. 0.87 g, 67% yield.

^l^H-NMR (300 MHz, CDCl_3_): δ 8.43 (s, 1H), 7.69 (d, *J* = 7.1 Hz, 2H), 7.52–7.59 (dd, *J* = 6.5 Hz, *J* = 9.0 Hz, 2H), 7.34 (t, *J* = 7.4 Hz, 2H), 7.03–7.28 (m, 12H), 6.61 (m, 1H), 4.18–4.33 (m, 2H), 4.01–4.18 (m, 4H), 3.88–4.01 (m, 2H), 3.01–3.24 (m, 2H), 2.45–2.69 (m, 4H), 1.93–2.16 (m, 4H).

^13^C-NMR (75 MHz, CDCl_3_): δ 173.2, 156.5, 144.3, 144.0, 141.7, 140.0, 139.9, 135.6, 132.4, 129.0, 128.7, 128.6, 128.2, 127.5, 126.8, 125.5, 125.4, 120.4, 67.2, 54.4, 49.6, 47.5, 47.1, 34.3, 32.8, 32.7, 31.55, 31.45, 26.6, 25.4, 25.3.

MS (MALDI-TOF) m/z 614.30[M]^+^.

### Synthesis of N (*α*)-[(9*H*-Fluoren-9-ylmethoxy) carbonyl]-N(*π*)-(7-phenylheptyl)-L-histidine methyl ester (Compound C3)

A solution of 7-phenyl-1-heptanol (526 µL, 2.63 mmol) and diisopropylethylamine (DIEA) (458 µL, 1.11 mmol) in DCM (15 mL), added dropwise over 10 min, was added to a stirred solution of triflic anhydride (443 µL, 1.11 mmol) in DCM (15 mL) under Ar at −75°C. Stirring was continued at −75°C (20 min). Then, a solution of FmocHis(Trt)-OMe (1.5 g, 2.37 mmol) in DCM (20 mL) was added dropwise, and the mixture was allowed to gradually warm to room temperature over a period of 16 h. The mixture was quenched using aqueous NaHCO_3_ and stirred vigorously (30 min). The organic layer was diluted with DCM, washed with aqueous NaHCO_3_ and brine, dried (MgSO_4_), and concentrated to viscous oil. Trifluoroacetic acid (1.68 mL, 23.70 mmol) and TIS (507 µL, 2.48 mmol) were added to a solution of the resulting gum in DCM (15 mL), and the mixture was stirred at room temperature until the reaction was complete, as shown by TLC (2 h). The solvent was removed in vacuo, and the residue was purified by silica gel–column chromatography using 1–5% MeOH in DCM to provide the product as a colorless gum (0.87 g, 65% yield).

^l^H-NMR (300 MHz, CDCl_3_): δ 8.48 (s, 1H), 7.77 (d, *J = *7.5 Hz, 2H), 7.57 (d, *J* = 7.0 Hz, 2H), 7.16–7.43 (m, 9H), 6.86 (s, 1H), 4.39 (m, 1H), 3.79–4.30 (m, 5H), 3.69 (s, 3H), 3.16 (s, 2H), 2.61 (t, *J* = 7.4 Hz, 2H), 1.67–1.86 (m, 2H), 1.47–1.66 (m, 2H), 1.32 (s, 6H).

MS (MALDI-TOF) m/z 566.14 [M]^+^.

### Synthesis of N (*α*)-[(9*H*-Fluoren-9-ylmethoxy) carbonyl]-N(*π*)-(7-phenylheptyl)-L-histidine (Compound D3)

The 1∶1 mixture of 2 N HCl (20 mL) and 1,4-Dioxane (20 mL) solution was added to the C3 (0.64 g, 1.13 mmol), and reflux occurred at 100°C for 3 h. The mixture was brought to room temperature, and the solvent was removed by Rota vapor. The resulting aqueous mixture was extracted with DCM, washed with brine, dried (MgSO_4_), and concentrated to viscous oil. The organic extract was dried (MgSO_4_) and concentrated in vacuo, and the residue was purified by silica gel–flash chromatography from 5% to 20% MeOH in DCM to provide the acid as a light yellow gum (0.50 g, 80% yield).

^l^H-NMR (300 MHz, CDCl_3_): δ 8.41 (s, 1H), 7.72 (d, *J* = 7.0 Hz, 2H), 7.46–7.63 (m, 2H), 7.30–7.43 (m, 2H), 7.07–7.40 (m, 9H), 6.51 (m, 1H), 4.56 (m, 1H), 4.22–4.37 (m, 2H), 4.11(m, 1H), 3.85–4.10 (m, 2H), 3.13–3.38 (m, 2H), 2.43–2.60 (m, 2H), 1.60–1.77 (m, 2H), 1.40–1.59 (m, 2H), 1.20 (s, 6H).

^13^C-NMR (75 MHz, CDCl_3_): δ 176.1, 156.1, 144.4, 144.0, 143.1, 141.6, 134.7, 131.7, 128.7, 128.6, 128.1, 127.4, 125.9, 125.8, 120.4, 120.0, 66.9, 55.8, 47.4, 46.9, 36.2, 31.7, 30.4, 29.6, 29.5, 29.3, 26.7.

MS (MALDI-TOF) m/z 552.12 [M]^+^.

### AMP: (WR)_3_-NH_2_

The purity of the crude product was 95% (C_18_ RP-HPLC). White powder; RP-HPLC R_t = _18.53 min (gradient B: 5–90%/30 min); MS (MALDI-TOF) m/z 1044.39 [M + H]^+^.

### AMP: (WR)_2_-NH_2_

The purity of the crude product was 95% (C_18_ RP-HPLC). White powder; RP-HPLC R_t_ = 17.27 min (gradient B: 5–90%/30 min); MS (MALDI-TOF) m/z 702.29 [M + H]^+^.

### AMP: RWR-NH_2_

The purity of the crude product was 95% (C_18_ RP-HPLC). White powder; RP-HPLC R_t_ = 13.62 min (gradient B: 5–90%/30 min); MS (MALDI-TOF) m/z 516.29 [M + H]^+^.

### Tetrameric HDAMP: DRDR-amide (where D = 1, histidine derivative)

The purity of the crude product was 95% (C_18_ RP-HPLC). White powder; RP-HPLC R_t_ = 37.7 min (linear gradient B, 0–90%/60 min); MS (MALDI-TOF) m/z 1300.96 [M]^+^.

### HDAMP-1, RDR-amide (where D = 1, histidine derivative)

The purity of the crude product was 95% (C18 RP-HPLC). White powder; RP-HPLC R_t_ = 29.21 min (linear gradient B, 0–90%/60 min); MS (MALDI-TOF) m/z 815.60 [M]^+^.

### HDAMP-2, RDR-amide (where D = 2, histidine derivative)

The purity of the crude product was 95% (C_18_ RP-HPLC).White powder; RP-HPLC R_t_ = 21.32 min (linear gradient B, 0–90%/60 min); MS (MALDI-TOF) m/z 703.50 [M]^+^.

### HDAMP-3, DR-amide (where D = 1, histidine derivative)

The purity of the crude product was 95% (C_18_ RP-HPLC). White powder; RP-HPLC R_t_ = 31.05 min (linear gradient B, 0–90%/60 min); MS (MALDI-TOF) m/z 659.57 [M]^+^.

### HDAMP-4, RDR-amide (where D = 3, histidine derivative)

The purity of the crude product was 95% (C_18_ RP-HPLC). White powder; RP-HPLC R_t_ = 21.77 min (linear gradient B, 0–90%/60 min); MS (MALDI-TOF) 641.42 [M + H]^+^.

### HDAMP-5, RDR-amide (where D = 1, histidine derivative, R = D-Arg)

The purity of the crude product was 95% (C_18_ RP-HPLC). White powder; RP-HPLC R_t_ = 28.91 min (linear gradient B, 0–90%/60 min); MS (MALDI-TOF) 816.66 [M]^+^.

### HDAMP-6, DR-amide (where D = 1, histidine derivative, R = D-Arg)

The purity of the crude product was 95% (C_18_ RP-HPLC). White powder; RP-HPLC R_t_ = 30.78 min (linear gradient B, 0–90%/60 min); MS (MALDI-TOF) 659.62 [M]^+^.

### FITC-labeled HDAMP-1

The purity of the crude product was 90% (C_18_ RP-HPLC). R_t_ = 41.35 (linear gradient, 0–90% B, 60 min); MS (MALDI-TOF) m/z = 1205.72 [M+H]^+^.

### Antimicrobial activity (MIC)

The antimicrobial activity of the peptides against two Gram-positive bacterial strains and two Gram-negative bacterial strains was examined using the broth microdilution method in sterile 96-well plates. Aliquots (100 µL) of a bacterial suspension at 2×10^6^ colony-forming units (CFU)/mL in 1% peptone were added to 100 µL of the peptide solution (serial two-fold dilutions in 1% peptone). After incubation for 18−20 h at 37°C, bacterial growth inhibition was determined by measuring the absorbance at 600 nm with a Microplate Autoreader EL 800 (Bio-Tek Instruments, VT). The minimal inhibitory concentration (MIC) was defined as the minimum peptide concentration inhibited bacteria growth. Two types of Gram-positive bacteria (*S. epidermidis* [KCTC 1917] and *S. aureus* [KCTC 1621]) and two types of Gram-negative bacteria (*E. coli* [KCTC 1682] and *P. aeruginosa* [KCTC 1637]) were procured from the Korean Collection for Type Cultures (KCTC) at the Korea Research Institute of Bioscience and Biotechnology (KRIBB). MRSA samples (CCARM 3089, CCARM 3090, and CCARM 3095) were obtained from the Culture Collection of Antibiotic-Resistant Microbes (CCARM) at Seoul Women's University (Seoul, Korea).

### Ethics Statement

This study was approved by the institutional ethics committee, and all healthy donors provided written informed consent before treatment. Consequently, we processed the blood samples according to the ethical standards of the Institutional Ethics Committee of Chosun University and according to the checklist for ethical consideration of cytotoxicity studies (https://www.cre.or.kr/article/policy/1382313). The collection of the human red blood cells (RBCs) from healthy donors was conducted at the Chosun University Hospital in Kwangju (Republic of Korea). Moreover, this study received ethics approval from the Institutional Ethics Committee of Chosun University. The authors of this article were blinded to all personal data from the donors, and all blood donors remained anonymous. All procedures were carried out according to rules provided by the Institutional Ethics Committee of Chosun University. Samples of blood were obtained from five healthy donors. The samples were stored immediately at 4°C until needed [41].

### Hemolytic activity

Hemolytic activity of peptides was tested against hRBCs. Fresh hRBCs were washed three times with phosphate-buffered saline (PBS; 35-mM phosphate buffer containing 150 mM of NaCl, pH 7.4) by centrifugation for 10 min at 1000×g and then re-suspended in PBS. The peptide solutions (serial two-fold dilutions in PBS) were then added to 100 µL of hRBCs in PBS to give a final volume of 200 µL and a final erythrocyte concentration of 4% (v/v). The resulting suspension was incubated with agitation for 1 h at 37°C. The samples were then centrifuged at 1000×g for 5 min, and hemoglobin release was monitored by measuring the absorbance of the supernatant at 405 nm. No hemolysis (blank) and 100% hemolysis controls consisted of hRBCs suspended in PBS and 0.1% Triton X-100, respectively. Percent hemolysis was calculated using the following equation:
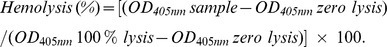



### Quantification of nitrite production in LPS-stimulated RAW264.7 cells

Nitrite accumulation in culture media was used as an indicator of NO production [46]. RAW264.7 cells were plated at a density of 5×10^5^ cells/mL in 96-well culture plates, and were stimulated with LPS (20 ng/mL) from *E. coli* O111:B4 (Sigma) in the presence or absence of peptides for 24 h. Isolated supernatant fractions were mixed with an equal volume of Griess reagent (1% sulfanilamide, 0.1% naphthylethylenediamine dihydrochloride, and 2% phosphoric acid) and incubated at room temperature for 10 min. Nitrite production was determined by measuring absorbance at 540 nm and converted to nitrite concentrations by reference to a standard curve generated with NaNO_2_.

### Quantification of TNF-α production by ELISA in LPS-stimulated RAW 264.7 cells

An antibody against mouse tumor necrosis factor-α (mTNF-α) was immobilized on immuno-plates by incubating with 0.2–0.8 µg/mL solutions of antibody in PBS overnight at room temperature. Plates were washed once with PBS [0.1% Tween 20 (PBST)] and blocked by incubating with 200 µL of blocking solution [3% bovine serum albumin (BSA), 0.02% NaN_3_ in PBS] overnight at room temperature. The supernatants from LPS-stimulated RAW264.7 cells co-incubated with serial-diluted peptide for 18 h were added to the wells of pre-coated plates and incubated for 2 h at room temperature. After washing plates three times with PBST, biotinylated anti-mTNF-α antibody (0.4 µg/mL), diluted in 0.1% BSA, was added, and plates were incubated for 2 h. Plates were then washed three times with PBST and further incubated with streptavidin peroxidase (0.3 µg/mL) diluted in PBS. After washing, SureBlue 3,3′,5,5′-tetramethylbenzidine peroxidase substrate (Kirkegaard & Perry Laboratories, Inc., Gaithersburg, MD) was added. The enzyme reaction was allowed to proceed at room temperature for color development, and was stopped by adding 100 µL of 1 M H_2_SO_4_. The absorbance at 450 nm was detected using a microplate reader. All values represent the mean ± S.D. of at least three independent experiments [47].

### LPS-neutralizing activity

The ability of the peptides to neutralize or inhibit LPS was assessed using a commercially available LAL assay kit (Kinetic-QCL 1000 kit; BioWhittaker, Walkersville, MD, USA). Briefly, 25 µL of serially diluted peptide was added in duplicate to 25 µL of *E. coli* O55:B5 LPS containing 3.0 U/mL endotoxin for 30 min at 37°C, followed by incubation with 50 µL of amoebocyte lysate reagent for 10 min. Absorbance at 405 nm was measured 10 and 16 min after the addition of 100 µL of the chromogenic substrate, Ac-Ile-Glu-Ala-Arg-p-nitroanilide. The amount of non-bound LPS was extrapolated from a standard curve, and the percentage of inhibition was calculated as: [(amount of free LPS in control samples) − (amount of free LPS in test samples)] ×100/amount of free LPS in control samples.

### Protease stability by radial diffusion assay

*E. coli* (KCTC 1682) was grown overnight for 18 h at 37°C in 10 mL of LB broth, and then 10 µl of this culture was inoculated into 10 mL of fresh LB and incubated for an additional 3 h at 37°C to obtain mid-logarithmic-phase organisms. For the radial diffusion assay method, a bacteria suspension (2×10^6^ CFU/mL in LB) was mixed with 0.7% agarose. The mixture was poured into a 10-cm petri dish after rapidly dispersing. Five microliters of an aqueous peptide stock solution (10 mg/mL) were added to 25 µL of the 0.2-µg/mL trypsin solution in PBS, respectively, and incubated at 37°C for 6 h. The reaction was stopped by freezing with liquid nitrogen, and then 10-µL aliquots were placed in each circle paper (≈6 mm in diameter) put on the agarose plates and then incubated at 37°C overnight. The diameters of the bacterial clearance zones surrounding the circle paper were measured for the quantitation of inhibitory activities.

### Protease stability by RP HPLC

Five microliters of an aqueous peptide stock solution (10 mg/mL) were added to 25 µL of the 0.2-µg/mL trypsin solution in PBS, respectively, and incubated at 37°C for 0, 0.5, 1.0, 2.0, and 4 h. The 5-µL solution was taken out at each time interval and immediately frozen with liquid nitrogen. Individually thawed samples were loaded onto a C_18_ reverse-phase column for HPLC analysis (5 µm; 4.6 mm ×250 mm; Vydac). Peptides were eluted by a 60-min linear gradient of 0–90% (v/v) acetonitrile in water containing 0.05% (v/v) trifluoroacetic acid with a flow rate of 1 mL/min and detection of 224 nm.

### Morphological changes of bacteria after HDAMP addition

Morphological changes of a Gram-negative bacterial strain (*E. coli* [KCTC 1682]) and a Gram-positive bacterial strain (*S. aureus* [KCTC 1621]) after the addition of HDAMPs (HDAMP-1, HDAMP-5, and HDAMP-6) were analyzed using TEM. The bacterial culture at 2×10^6^ CFU/mL in LB media was washed three times in PBS via a series of centrifugation at 10,000× g, for 5 min, and re-suspension. One-hundred microliters of HDAMPs in PBS were added to an equal volume of bacterial suspension to a final concentration at ×10 MIC. Following the addition of HDAMPs, the samples were incubated for 1 h at 37°C. Bacterial cell pellet after centrifugation was re-suspended in 20 µl PBS for TEM specimen preparation. Five microliters of sample solution were loaded onto a carbon film-coated TEM grid, which was rendered hydrophilic by glow discharge. After 90 s, excess sample solution was washed off with distilled water. Five microliters of 1% uranyl acetate were loaded onto the grid for negative staining for 1 min, and excess stain solution was blotted using a piece of filter paper. Samples were imaged using a Tecnai G^2^ Spirit electron microscope (FEI) equipped with a lanthanum hexaboride (Lab_6_) gun, operating at 120 kV. Images were recorded using the Ultrascan 4000 charge-coupled device (CCD) camera (Gatan).

### Confocal laser-scanning microscopy

*E. coli* (KCTC 1682) and *S. aureus* (KCTC 1621) cells in the mid-logarithmic phase were harvested by centrifugation, washed three times with a 10-mM phosphate buffer saline, pH 7.4. Bacteria (10^7^ CFU/mL) cells were incubated with FITC-labeled peptides (5 µg/mL) at 37°C for 30 min. After being incubated, the bacterial cells were pelleted down and washed three times with the 10 mM sodium phosphate buffer, pH 7.4, and immobilized on a glass slide. The FITC-labeled peptides were observed with a Zeiss Axioplan 2 optical microscope (Japan). Fluorescent images were obtained with a 488-nm band-pass filter for excitation of FITC.

### Membrane depolarization

The cytoplasmic membrane depolarization activity of peptides was determined by the membrane potential–sensitive dye, diSC_3_-5. Briefly, *S. aureus* was grown at 37°C with agitation to the mid-log phase (OD_600_ = 0.4), and was harvested by centrifuge. The cells were washed twice with washing buffer (20 mM glucose, 5 mM HEPES, pH 7.4) and re-suspended to an OD_600_ of 0.05 in a similar buffer containing 0.1 M KCl. Thereafter, the cells were incubated with 20 nM of DiSC_3_-5 until a stable reduction of fluorescence was achieved, indicating the incorporation of the dye into the bacterial membrane. Membrane depolarization was then monitored by observing the change in the fluorescence emission intensity of the membrane potential–sensitive dye, DiSC_3_-5 (*λ*_ex._ = 622 nm, *λ*_em_ = 670 nm), after the addition of peptides. Full dissipation of the membrane potential was obtained by adding gramicidin D (final concentration of 0.2 nM).

### Dye leakage

Calcein-entrapped LUVs (large unilamellar vesicles) composed of PE/PG (7∶3, w/w) were prepared by vortexing the dried lipid in dye buffer solution (70 mM calcein, 10 mM Tris, 150 mM NaCl, 0.1 mM EDTA, pH 7.4). The suspension was subjected to 10 frozen-thaw cycles in liquid nitrogen and extruded 21 times through polycarbonate filters (two stacked 100-nm pore-size filters) with a LiposoFast extruder (Avestin, Inc. Canada). Untrapped calcein was removed by gel filtration on a Sephadex G-50 column. Calcein leakage from LUVs was monitored at room temperature by measuring fluorescence intensity at an excitation wavelength of 490 nm and emission wavelength of 520 nm on a model RF-5301PC spectrophotometer. Complete dye release was obtained using 0.1% Triton X-100.

Abbreviations: AMP, antimicrobial peptide; MIC, minimal inhibitory concentration; MRSA, methicillin-resistant *Staphylococcus aureus*

## Supporting Information

Figure S1 **Concentration–response curves of percent hemolysis of the peptides against human red blood cells.** Peptides are indicated as follows: (WR)_3_-NH_2_ (•), (WR)_2_-NH_2_ (○), RWR-NH_2_ (▾), HDAMP-1 (▽), HDAMP-2 (▪), HDAMP-3 (□), HDAMP-4 (⧫), HDAMP-5 (◊), HDAMP-6 (▴), and LL-37(△).
(TIF)
