# Supplementary figures and images for: De Novo Design and Synthesis of Ultra-Short Peptidomimetic Antibiotics Having Dual Antimicrobial and Anti-Inflammatory Activities
Source: PLoS One. 2013 Nov 26;8(11):e80025. doi: 10.1371/journal.pone.0080025 (PMC3841161; doi:10.1371/journal.pone.0080025)

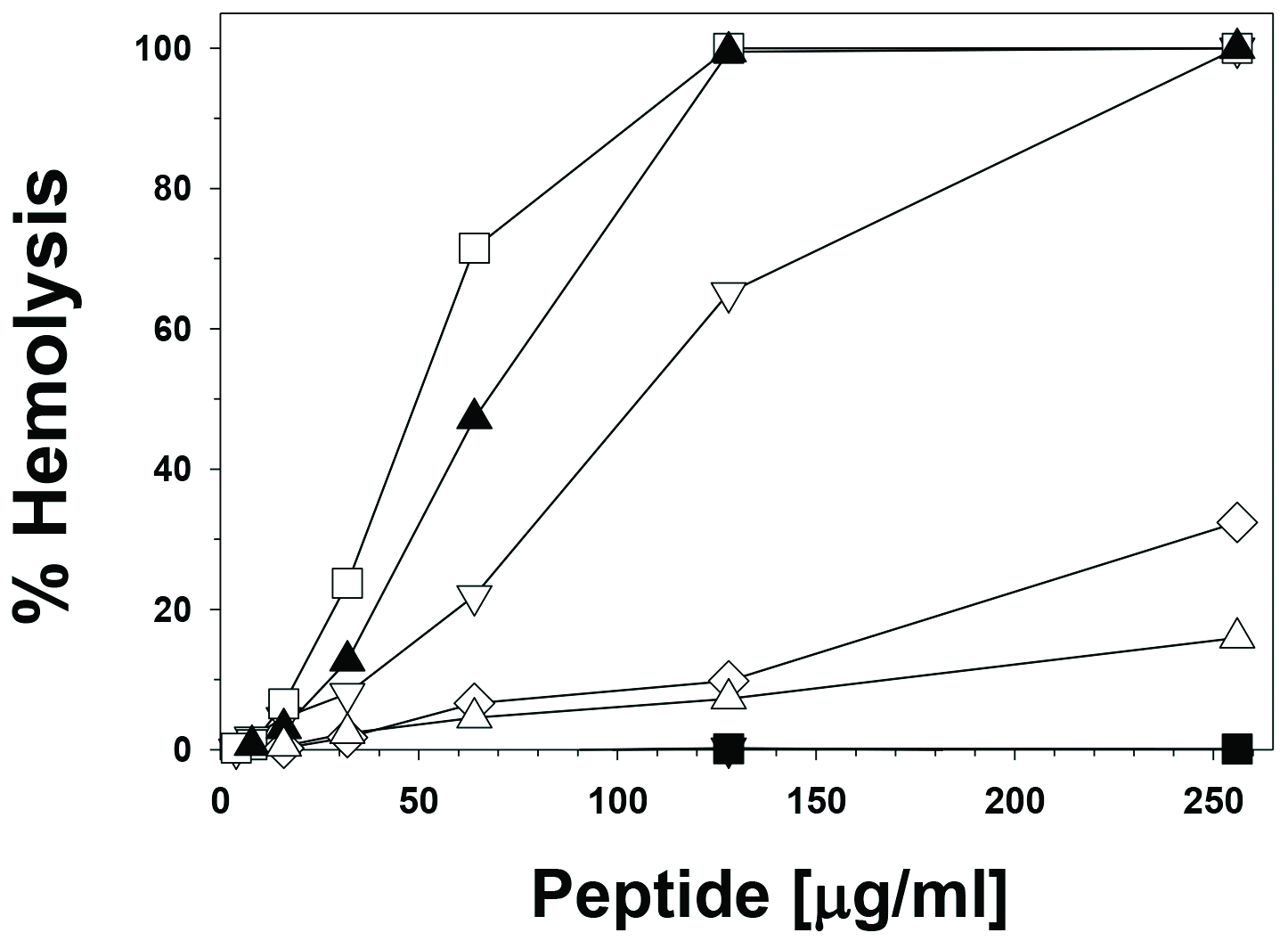

Supplement: Figure S1 — Concentration–response curves of percent hemolysis of the peptides against human red blood cells. Peptides are indicated as follows: (WR)3-NH2 (•), (WR)2-NH2 (○), RWR-NH2 (▾), HDAMP-1 (▽), HDAMP-2 (▪), HDAMP-3 (□), HDAMP-4 (⧫), HDAMP-5 (◊), HDAMP-6 (▴), and LL-37(△). (TIF) [file pone.0080025.s001.tif]
